# Application of Low Coverage Genotyping by Sequencing in Selectively Bred Arctic Charr (*Salvelinus alpinus*)

**DOI:** 10.1534/g3.120.401295

**Published:** 2020-04-20

**Authors:** Christos Palaiokostas, Shannon M. Clarke, Henrik Jeuthe, Rudiger Brauning, Timothy P. Bilton, Ken G. Dodds, John C. McEwan, Dirk-Jan De Koning

**Affiliations:** * Department of Animal Breeding and Genetics, Swedish University of Agricultural Sciences, Box 7090, 750 07 Uppsala, Sweden,; ^†^ Invermay Agricultural Centre, AgResearch, Private Bag 50034, Mosgiel 9053, New Zealand,; ^‡^ Aquaculture Center North, Åvägen 17, 844 61 Kälarne, Sweden, and; ^§^ Department of Mathematics and Statistics, University of Otago, Dunedin 9054, New Zealand

**Keywords:** Arctic charr, genotyping by sequencing, selective breeding

## Abstract

Arctic charr (*Salvelinus alpinus*) is a species of high economic value for the aquaculture industry, and of high ecological value due to its Holarctic distribution in both marine and freshwater environments. Novel genome sequencing approaches enable the study of population and quantitative genetic parameters even on species with limited or no prior genomic resources. Low coverage genotyping by sequencing (GBS) was applied in a selected strain of Arctic charr in Sweden originating from a landlocked freshwater population. For the needs of the current study, animals from year classes 2013 (171 animals, parental population) and 2017 (759 animals; 13 full sib families) were used as a template for identifying genome wide single nucleotide polymorphisms (SNPs). GBS libraries were constructed using the PstI and MspI restriction enzymes. Approximately 14.5K SNPs passed quality control and were used for estimating a genomic relationship matrix. Thereafter a wide range of analyses were conducted in order to gain insights regarding genetic diversity and investigate the efficiency of the genomic information for parentage assignment and breeding value estimation. Heterozygosity estimates for both year classes suggested a slight excess of heterozygotes. Furthermore, F_ST_ estimates among the families of year class 2017 ranged between 0.009 – 0.066. Principal components analysis (PCA) and discriminant analysis of principal components (DAPC) were applied aiming to identify the existence of genetic clusters among the studied population. Results obtained were in accordance with pedigree records allowing the identification of individual families. Additionally, DNA parentage verification was performed, with results in accordance with the pedigree records with the exception of a putative dam where full sib genotypes suggested a potential recording error. Breeding value estimation for juvenile growth through the usage of the estimated genomic relationship matrix clearly outperformed the pedigree equivalent in terms of prediction accuracy (0.51 opposed to 0.31). Overall, low coverage GBS has proven to be a cost-effective genotyping platform that is expected to boost the selection efficiency of the Arctic charr breeding program.

Genomic information is being incorporated at an ever increasing rate in aquaculture breeding programs, guiding selection decisions (Yanez *et al.* 2015). Use of genome wide genetic markers has the potential to unravel the genetic factors that control traits of interest, increase the accuracy of estimated breeding values (Meuwissen *et al.* 2001) and optimize inbreeding management (D’Ambrosio *et al.* 2019). Current knowledge supported by both simulation (Sonesson and Meuwissen 2009) and real data (reviewed in Palaiokostas and Houston 2017; Lhorente *et al.* 2019) in a wide range of aquaculture species suggests that substantial improvements for key production traits can be obtained through use of genomic information. In particular, benefits are most pronounced for disease challenges, sex-limited-, late-in-life-, and post-slaughter traits.

Implementation of genomics in both animal and plant breeding programs is commonly performed through the usage of single nucleotide polymorphism (SNP) arrays (reviewed in Hickey *et al.* 2017) that typically contain tens or hundreds of thousands of individual assays. However, SNP arrays typically require considerable prior investment, their price per unit is expensive unless purchased in large volumes (in the magnitude of hundreds of thousands), and potentially suffer from SNP ascertainment bias (Lachance and Tishkoff 2013). Advancements in high throughput sequencing technologies offer cost effective alternatives in the form of reduced representation genomic DNA libraries (Davey *et al.* 2011). A wide range of library construction protocols have been developed like restriction-site associated DNA sequencing (RAD-seq; Baird *et al.* 2008) and genotyping by sequencing (GBS; Elshire *et al.* 2011). The main premise of GBS type methodologies is the reduction of the genome complexity via using restriction enzyme(s) of choice. As such, sequencing occurs only in size selected DNA fragments resulting from the enzymatic digestion.

The plethora of available type II restriction enzymes make the aforementioned platforms particularly flexible, allowing researchers to decide between dense or sparse genotyping strategies (Rochette *et al.* 2019). Furthermore, through barcode ligation, multiple animals can be pooled in a sequencing lane and subsequently de-multiplexed bioinformatically thus allowing significant cost reductions per animal unit (Palaiokostas *et al.* 2015; Brown *et al.* 2016; Robledo *et al.* 2017). Taking into account that high throughput sequencers have pre-determined costs and data output (sequencer model dependent) per sequencing run, it is apparent that a tradeoff exists between number of animals that can be utilized in a sequencing lane and the expected sequencing coverage per animal.

In contrast, an ideal scenario, particularly suitable for selective breeding purposes, will favor sequencing libraries that are produced with frequent enzymatic cutters (resulting in dense genotyping) and are composed from a multiplex including a large number of animals. Low coverage GBS offer an attractive solution that offers for both high SNP density and cost-effective genotyping (Dodds *et al.* 2015). GBS using low sequencing coverage has been successfully used for estimating genome wide linkage disequilibrium (Bilton *et al.* 2018a), constructing genetic maps (Bilton *et al.* 2018b; Chagné *et al.* 2018), parentage assignment (Dodds *et al.* 2019), predicting gender in animals (Bilton *et al.* 2019) and implementing genomic selection (Faville *et al.* 2018).

Arctic charr (*Salvelinus alpinus*) farming is a small but growing industry with ample margin for production scaling. Sweden is the second largest producer of Arctic charr worldwide with a production volume of approximately 1,310 tons (SCB 2017), while global production is estimated between 6,000 – 10,000 tons (Olk *et al.* 2019). An ongoing national breeding program for Arctic charr has been in place for approximately 40 years, resulting in an improved strain capable of reaching market size (600 - 800 g) one year earlier compared to wild stocks (Eriksson *et al.* 2010). The breeding strain originates from a landlocked population in lake Hornavan (Sweden) and no external germplasm has ever been included in the breeding nucleus. The Hornavan Arctic charr demonstrated superior growth capacity compared to other Arctic charr populations in Sweden and was thus selected for the establishment of a selective breeding program (Nilsson *et al.* 2010). Currently, selection candidates are chosen using best linear unbiased prediction (BLUP) methodology (Henderson 1975) for growth related traits. Typically, the breeding program has been based on 45 – 125 full sib families (Nilsson *et al.* 2010) reared in separate tanks until a size suitable (30 – 60 g) for identification using passive integrated transponders (PIT) tags. Thereafter the breeding candidates are reared communally in inland facilities.

Compared to the ample genomic information available in farmed salmonids like Atlantic salmon (*Salmo salar*) and rainbow trout (*Oncorynchus mykiss*), genomic resources for Arctic charr have only started to become available very recently (Nugent *et al.* 2017, 2019; Christensen *et al.* 2018). Nevertheless, no prior work has ever attempted to investigate the genetic diversity status and the potential for further improving the selectively bred Arctic charr strain in Sweden through the use of genome-wide genetic markers. The aim of the current study was to apply low coverage GBS in the aforementioned breeding strain using 930 animals from two-year classes (2013; 2017). Genome wide SNPs were identified and subsequently utilized for estimating genetic diversity metrics, verifying available parentage records and for estimation of breeding values through genomic BLUP (GBLUP).

## Materials and Methods

### Sample background

Animals used in this study originated from two discrete year classes (2013 and 2017) of the Swedish Arctic charr breeding program. The breeding nucleus is located in the facilities of Aquaculture Center North (ACN; Kälarne, Jämtland, Sweden) where selection is operated using non-overlapping generations of approximately 4 years each. As such, the 2017 year class was formed through artificial crosses among selected animals from the 2013 year class. In the current study, 171 animals from year class 2013 (selected based on DNA availability) and 759 animals from year 2017 were genotyped using GBS. Genotyped animals from year class 2017 originated from crosses between 11 sires and 13 dams. Due to the aforementioned breeding scheme the genotyped animals of 2017 year class consisted of 13 full-sib families and two pairs of paternal half-sib families. Family size ranged between 10 – 90 offspring.

According to available pedigree data, five sires and four dams from the genotyped animals of year class 2013 were the parents of eight genotyped families from year class 2017. In particular, two families had both their putative parents genotyped, six had only one parent genotyped (two families had only their dam and four families had only their sire genotyped) and the remaining five families had neither of their parents genotyped (Table 1). Moreover, upon PIT-tagging fin-clips were collected from each animal for DNA extraction and stored in absolute ethanol at -20°. The entire study was conducted in accordance to Swedish legislation for conducting animal research as described in the Animal Welfare Act 2018:1192 (ethics permit: 5.2.18 – 09859/2019).

**Table 1:** Pedigree information for genotyped animals from year class 2017

Family Id	Size	No genotyped parents	Sire	Dam	Paternal grandsire	Paternal granddam	Maternal grandsire	Maternal granddam
F1	63	1	S1	D1	GPS1	GPD1	GPS2	GPD2
F2	90	2	S2	D2	N/A	N/A	GPS3	GPD3
F3	34	1	S3	D3	GPS4	GPD4	GPS2	GPD2
F4	20	0	S4	D4	N/A	N/A	GPS5	GPD5
F5	62	0	S5	D5	GPS6	GPD6	GPS2	GPD2
F6	90	0	S6	D6	GPS7	GPD7	N/A	N/A
F7	61	1	S7	D7	GPS2	GPD2	GPS6	GPD6
F8	61	1	S7	D8	GPS2	GPD2	GPS8	GPD8
F9	90	2	S8	D9	GPS9	GPD9	GPS10	GPD10
F10	16	1	S8	D10	GPS9	GPD9	GPS8	GPD11
F11	90	0	S9	D11	GPS11	GPD12	GPS12	GPD13
F12	62	0	S10	D12	GPS13	GPD14	GPS5	GPD5
F13	20	1	S11	D13	GPS14	GPD15	GPS12	GPD13

### GBS library preparation and sequencing

A tissue plug of approximately 3 mm diameter from each fin clip was used for DNA extraction. Prior to extraction, the tissue was air dried overnight to remove all traces of ethanol and then DNA was extracted following a salt-based extraction protocol (Clarke *et al.* 2014). A subset of the DNA extracted samples was visually assessed via 1.0% agarose gel in order to ensure the existence of high molecular weight DNA. Quantification was performed using Picogreen (Quant-iT Picogreen dsDNA Reagent, Cat P11495, Life Technologies, Carlsbad, California, United States) fluorescence. Template DNA was digested with PstI (recognizing the CTGCA|G motif) and MspI (recognizing the C|CGG motif) restriction enzymes. Subsequent library preparation followed the method outlined in Elshire *et al.* (2011). Constructed libraries were size selected between 193 – 318 bp using a BluePippin (Sage Science). In total 10 GBS libraries were constructed containing 90 – 94 individuals each. Sequencing was performed in 5 lanes of an Illumina HiSeq 2500 using 100 cycles single end (SE) V4 chemistry.

### QC filtering and SNP identification

Reads of low quality (average phred33 score below 30) were discarded using trimmomatic v0.39 (Bolger *et al.* 2014). SNP identification was performed using the UNEAK, Tassel v3.0.174 software (Lu *et al.* 2013) using the following settings: *-UFastqToTagCountPlugin -e PstI-MspI*; *-UMergeTaxaTagCountPlugin -c 10*; *-UTagCountToTagPairPlugin -e 0.03*; *-UMapInfoToHapMapPlugin -mnMAF 0.05 -mxMAF 0.5 -mnC 0.1*. SNPs with mean coverage below 0.5X were discarded. Finally, SNPs with Hardy-Weinberg disequilibrium (observed frequency of the reference allele homozygote minus its expected value) below -0.05 were removed using the KGD v0.8.7 software.

### Genomic relationships

A genomic relationship matrix (GRM) was estimated using the KGD v0.8.7 software (https://github.com/AgResearch/KGD). The constructed GRM was the equivalent of G5, as described in Dodds *et al.* (2015). In short, an initial GRM using the SNPs passing the aforementioned QC filters was constructed (VanRaden 2008). Subsequently, the diagonals were corrected in order to account for sequence depth. Finally, the off-diagonal elements of the GRM were calculated using only SNPs that were scored in both of the corresponding individuals. The range of genomic relationships among full-sibs was inferred from the constructed GRM.

### Genetic diversity – population structure

Mean expected (He) and observed (Ho) heterozygosity were estimated separately for animals from year class 2013 and 2017 using the KGD v0.8.7 software. Heterozygosity metrics were estimated both on the raw scale and by taking into account the read depth. Moreover, F_ST_ values among the genotyped families of year class 2017 were estimated using the aforementioned software. Additionally, principal component analysis (PCA) was performed in order to investigate the population structure of the two separate year classes. PCA was conducted through applying the singular value decomposition (SVD) algorithm to the estimated GRM. Finally, aiming to investigate in more depth potential genetic clustering of the genotyped families (year class 2017), the discriminant analysis of principal components (DAPC) was performed (Jombart *et al.* 2010). PCA was initially applied in the constructed GRM as previously mentioned. A cross validation step was followed using the *xvalDapc* function to select the optimal number of PC for the DAPC. Thereafter, a discriminant analysis step was conducted using clusters determined from the principal components. The Bayesian Information Criterion (BIC) was used for selecting the optimal number of clusters (K) based on the elbow method (Jombart *et al.* 2010).

### Parentage assignment

SNPs passing QC filters were used for parentage assignment purposes. Assignment to most probable sire and dam was performed taking into account the sequence depth of the corresponding individuals as described in Dodds *et al.* (2019). Specifically, the excess mismatch rate (EMM) metric was considered for parentage verification using a maximum threshold of 0.025. Furthermore, successful assignment was accepted only when the relatedness (according to the GRM) among putative parents and offspring was above 0.30.

### Estimation of heritability for growth traits – breeding value accuracy

Heritability estimates of juvenile weight and length were obtained using the constructed GRM. Variance components were estimated using AIREMLF90 (Misztal *et al.* 2014) with the following animal model:

y=Xb+Zu+Tc+e (1)

where **y** is the vector of recorded phenotypes. **X**, **Z** and **T** are the incidence matrices relating phenotypes with fixed and random effects. **b** is the vector of the fixed effects (intercept and age), **u** the vector of random animal effects ∼*N*(0, **G**$\sigma_{g}^{2}$) with **G** corresponding to the genomic relationship matrix (Dodds *et al.* 2015), $\sigma_{g}^{2}$ the additive genetic variance, **c** the vector of random effect representing the full-sib common environmental effect due to rearing each family in separate tanks ∼ *N*(0, **I**$\sigma_{\text{c}}^{2}$), where $\sigma_{\text{c}}^{2}$ the corresponding variance, **e** the vector of residuals ∼*N*(0, **I**σ_e_^2^), **I** the identity matrix and $\sigma_{e}^{2}$ the residual variance.

The common full-sib effect was estimated using the following formula:

$$c^{2}=\frac{\sigma_{c}^{2}}{\sigma_{g}^{2}+\sigma_{\text{c}}^{2}+\sigma_{e}^{2}}$$,

Heritability for juvenile growth and length was estimated using the following formula:

$$h^{2}=\frac{\sigma_{g}^{2}}{\sigma_{g}^{2}+\sigma_{\text{c}}^{2}+\sigma_{e}^{2}}$$,

The prediction accuracy of genomic breeding values (GEBVs) was calculated and benchmarked against the accuracy of EBVs using traditional pedigree-based best linear unbiased prediction (BLUP) (Henderson 1975). Accuracy comparisons between BLUP and GBLUP were performed using a threefold cross validation scheme. For this, the PIT-tagged individuals were split into 3 groups (the number of animals within each group ranged between 241 – 263) with an equal family representation among groups. Each of these groups in turn was used as a validation set, while the other two groups were used in the training set. The cross validation procedure was repeated 10 times in order to reduce random sampling effects.

GEBVs were estimated with GBLUP (Meuwissen *et al.* 2001) using the BLUPF90 suite (Misztal *et al.* 2014) updated for genomic analyses (Aguilar *et al.* 2011). Pedigree-based BLUP was applied to calculate breeding values using the same software. The general form of the fitted models was as in equation (1).

The prediction accuracy was approximated as:

r=correlation(GEBV,y)/h, (2)

where **y** is the vector of recorded phenotypes, (G)EBV is the vector of (genomic) estimated breeding values and h is the square root of the previously estimated heritability. In order to have meaningful comparisons between BLUP and GBLUP we used in both cases the heritability estimated through the genomic relationship matrix.

## Data availability

Obtained sequences are available in the form of FASTQ files from NCBI repository under project ID PRJNA607181. Information regarding the utilized barcode and the sequencing lane of each library is available in supplementary file S1. Genotypic and pedigree information is available in the supplementary files S2 and S3. Phenotypic information for the genotyped animals of 2017 year class is available in the supplementary file S4. Supplemental material available at figshare: https://doi.org/10.25387/g3.12151725.

## Results

### Dataset filtering

**Figure 1:**
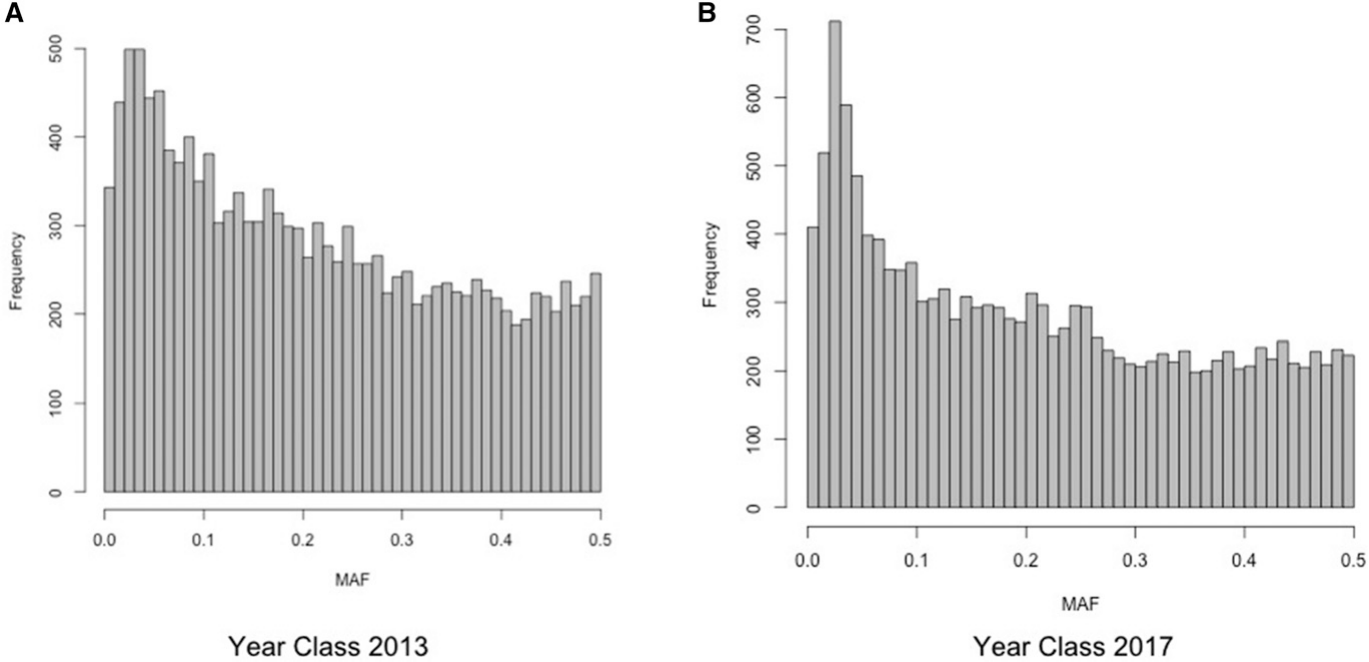
Distribution of minor allele frequency. A) Year class 2013. B) Year class 2017.

More than 1.35 billion SE reads were produced. Approximately 97% of the above reads (∼1.32 billion) had an average phred33 score above 30 and were kept for downstream analysis. Reads without the expected enzymatic cut site and no recognizable barcode were discarded, resulting in ∼964 million SE reads. Thereafter reads were merged in 662,978 unique tags that were used as a template for SNP identification. We removed 2 animals (year class 2013) that were genotyped insufficiently (mean coverage < 0.1X). In total 17,652 SNPs were identified across 928 genotyped individuals. 70 SNPs with mean coverage below 0.5X were removed. The mean SNP coverage across animals was approximately 5X. Finally, 3,134 SNPs did not fulfill the chosen Hardy-Weinberg equilibrium (HWE) threshold and were removed. Overall, the final dataset was comprised of 14,518 SNPs (Figure 1) genotyped across 928 animals.

### Genomic relationships

**Figure 2:**
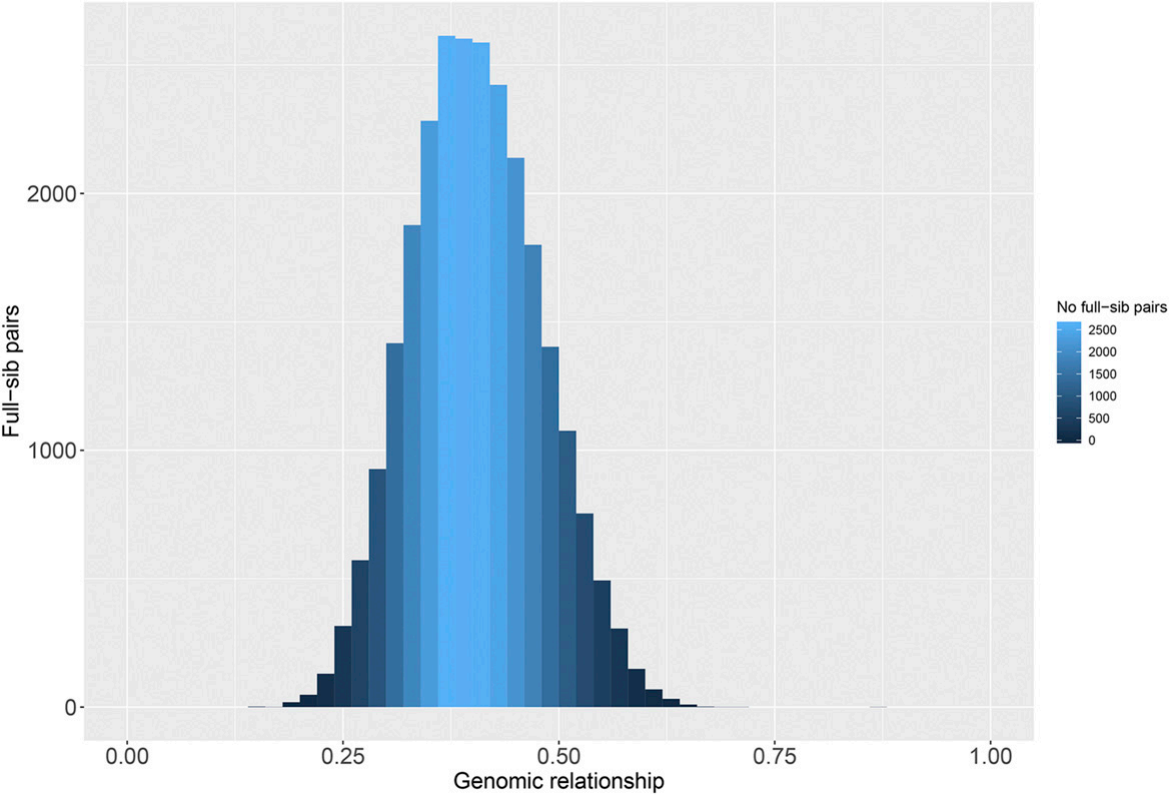
Realized relationships among full-sib pairs.

Among the genotyped full-sib pairs, six animals from four families had low genomic relationships with their full-sibs (below 0.10) and were removed from subsequent analysis. The mean genomic relationship among all full-sib pairs was 0.42 (Figure 2). Furthermore, since the estimated genomic relationship matrix was non positive definite, a bending approach was applied where a predefined threshold of 0.001 was used to replace eigenvalues below the aforementioned threshold (Schaeffer 2014) constituting the obtained matrix invertible. The ‘bent’ GRM was used for estimating GEBVs using GBLUP (Figure S1).

### Genetic diversity – population structure

Heterozygosity metrics were estimated both in the raw scale and by taking into account the read depth of the corresponding SNP alleles. Higher estimates of heterozygosity were found in the 2013 year class. Observed heterozygosity (H_o_) when the read depth was not taken into account was 0.21 and 0.19 in year class 2013 and 2017 respectively, while the corresponding estimates of expected heterozygosity (H_e_) were 0.19 and 0.17. When the read depth was taken into account the H_o_ was found equal to 0.32 and 0.31 for year class 2013 and 2017 respectively. Moreover, the H_e_ for the above year classes was 0.29 and 0.28. F_ST_ based calculations were performed among the families of year class 2017. The median F_ST_ ranged between 0.009 – 0.066, with the lowest estimate being observed between families 7 and 8 and the highest between families 7 and 11 (Table 2).

**Table 2:** Median Fst values (14,518 SNPs) among families

	F1	F2	F3	F4	F5	F6	F7	F8	F9	F10	F11	F12	F13
**F1**	—	0.063	0.031	0.041	0.039	0.046	0.037	0.043	0.045	0.032	0.056	0.053	0.036
**F2**		—	0.049	0.035	0.056	0.060	0.057	0.034	0.052	0.027	0.067	0.056	0.033
**F3**			—	0.049	0.028	0.041	0.035	0.038	0.032	0.038	0.046	0.046	0.046
**F4**				—	0.043	0.017	0.042	0.044	0.029	0.050	0.039	0.027	0.052
**F5**					—	0.055	0.012	0.038	0.040	0.034	0.063	0.056	0.039
**F6**						—	0.059	0.051	0.047	0.029	0.063	0.045	0.029
**F7**							—	0.009	0.040	0.032	0.066	0.058	0.043
**F8**								—	0.048	0.029	0.059	0.063	0.043
**F9**									—	0.010	0.048	0.046	0.027
**F10**										—	0.032	0.040	0.049
**F11**											—	0.060	0.029
**F12**												—	0.040
**F13**													—

**Figure 3:**
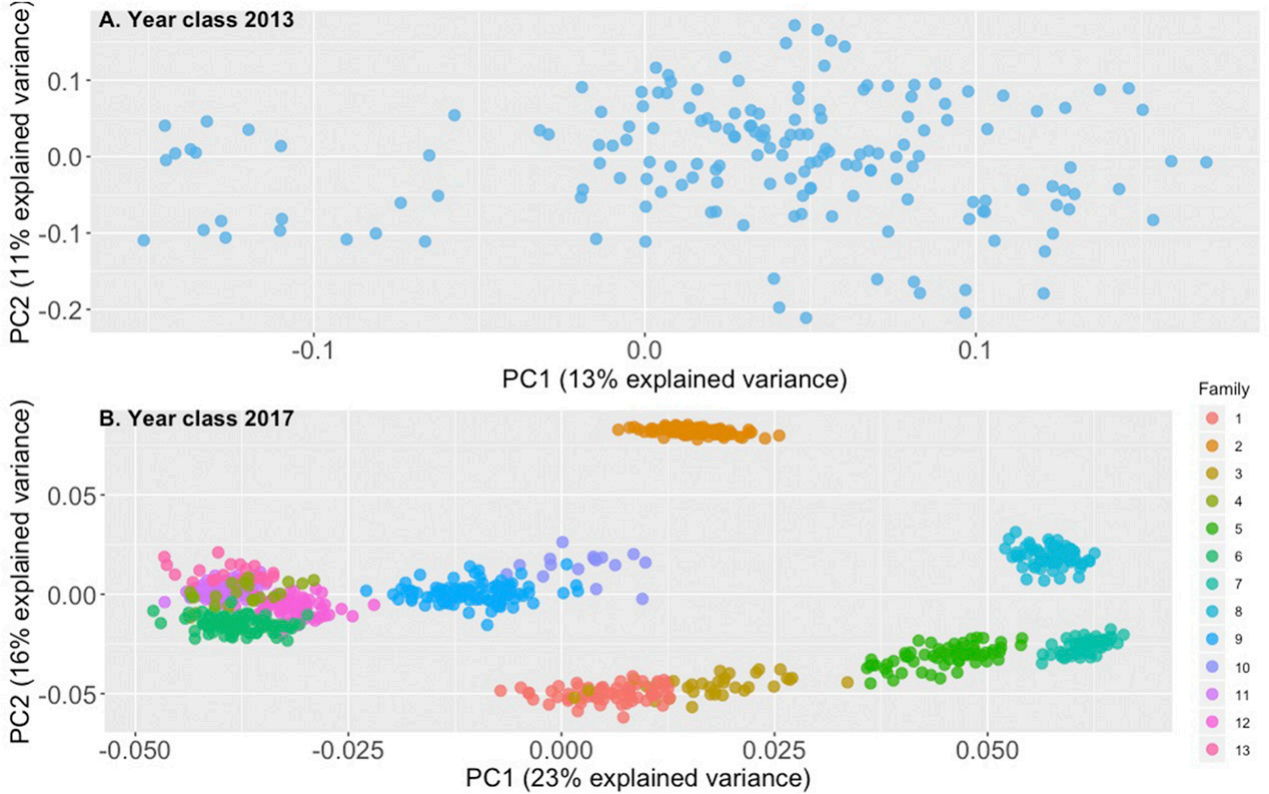
A) Principal component analysis for the 2013 year class. B) Principal component analysis for the 2017 year class.

**Figure 4:**
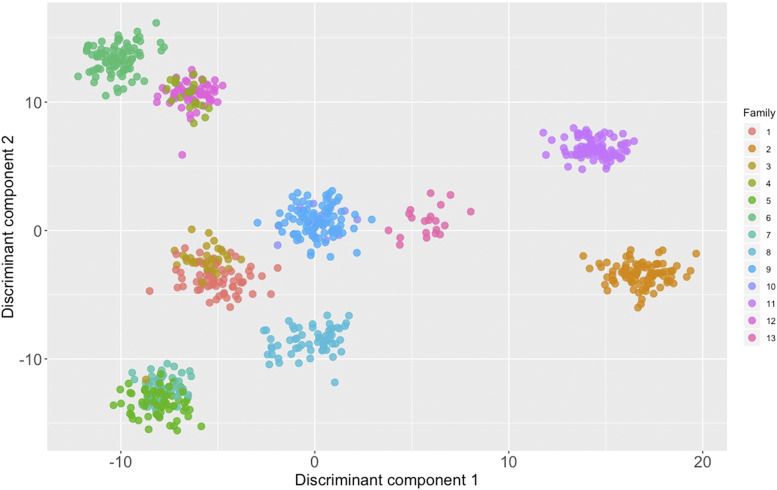
Discriminant analysis of principal components in the 2017 year class.

Principal component analysis was used as a dimensionality reduction technique both for visualization purposes and to infer potential clustering among genotyped animals. Regarding animals from the 2013 year class the first and second principal components accounted for 13% and 11% of the total variance respectively (Figure 3). Moreover, in the case of animals from the 2017 year class the first and second principal components accounted for 23% and 16% of the total variance (Figure 3). DAPC was applied in order to decipher the genetic structure of 2017 animals and identify potential clusters (Figure 4). Cross validation suggested that the optimal number of principal components for clustering was 20 (Figure S2). Moreover, according to the obtained BIC the optimal K was found to be 13 (Figure S3). Results from DAPC were generally in accordance with the preliminary structure suggested by PCA. According to DAPC families 9 and 10 were indistinguishable. The aforementioned families shared the same sire. A similar pattern was observed between families 5 and 7, who had the same grandsires and granddams (Table 1). On the other hand, families 2 and 11 formed unique clusters (Figures 3, 4).

### Parentage assignment

Parentage assignment was performed for the eight families where at least one parent (from year class 2013) was genotyped. The results suggested that the identity of the putative dam in family 2 was most probably incorrectly recorded. On the other hand, the putative sire of the same family was confirmed by 98.9% of the offspring. Regarding the other seven families, the genotypic data verified the available parentage pedigree records. Between 97.8–100% of offspring in each family were in accordance with the pedigree data. EMM was below 1% for all families apart from family 2 were EMM for the putative dam was approximately 5.4% (Table 3).

**Table 3:** Verification of suggested parentage from pedigree records

Family Id	Mean Dam relationship	Mean Dam EMM % (SE)	Mean Sire relationship	Mean Sire EMM % (SE)	Dam verified offspring (%)	Sire verified offspring (%)
1	N/A	N/A	0.41	0.24 (0.08)	N/A	98.4
2	0.01	5.39 (0.04)	0.41	0.23 (0.04)	0	98.9
3	N/A	N/A	0.39	0.33 (0.13)	N/A	97.1
7	0.35	0.44 (0.03)	N/A	N/A	100	N/A
8	0.37	0.25 (0.04)	N/A	N/A	100	N/A
9	0.38	0.34 (0.08)	0.32	0.21 (0.04)	97.8	97.8
10	N/A	N/A	0.37	0.04 (0.08)	N/A	100
13	N/A	N/A	0.50	0.21 (0.03)	N/A	100

EMM refers to excess mismatch rate.

### Estimation of heritability for growth traits – breeding value accuracy

The estimated heritability for juvenile weight and length was 0.21 (SE 0.07) and 0.19 (SE) respectively. The corresponding full sib effect for weight and length was 0.11 (SE 0.08) and 0.12 (SE 0.08). Genetic correlation between weight and length was 0.94 (SE 0.13). For the subsequent breeding value accuracy estimation only the weight was used due to the high correlation between the two traits. The mean accuracy using GBLUP was 0.51, while the corresponding accuracy using pedigree BLUP was 0.31. In the case of GBLUP the obtained accuracy of the cross validation scheme used ranged between 0.42 – 0.56, while in the case of pedigree BLUP accuracy ranged between 0.28 – 0.35 (Table 4).

**Table 4:** Accuracy comparison between GBLUP and PBLUP using threefold cross validation (10 replicates)

Validation Id	Group size	GBLUP accuracy (SE)	PBLUP accuracy (SE)
1	249	0.56 (0.03)	0.35 (0.03)
2	241	0.54 (0.03)	0.29 (0.03)
3	263	0.42 (0.04)	0.28 (0.03)
**Overall**	**753**	**0.51 (0.03)**	**0.31 (0.03)**

## Discussion

Genomic technologies can be utilized to estimate relationship values among the selection candidates at a higher resolution than a traditional pedigree-based analysis would allow. Relationships based on pedigree records will assign the same value to all full-sib members of individual families, making it impossible to utilize the within family variance for selection. Therefore, the information obtained through genomics can result in an increased accuracy of breeding values and a more efficient management of inbreeding.

A plethora of recent studies have already highlighted the advantages of including genomic information in aquaculture breeding programs (Robledo *et al.* 2018; Houston and Macqueen 2019; Yoshida *et al.* 2019; Boison *et al.* 2019; Saura *et al.* 2019; Vallejo *et al.* 2019; Barría *et al.* 2018; Tsairidou *et al.* 2019; Joshi *et al.* 2020). Nevertheless, genotyping related costs often limit the implementation of genomic technologies in practice. Cost effective genotyping approaches like GBS appear particularly suitable for small – medium sized breeding programs as is the case of Arctic charr breeding in Sweden.

Low coverage GBS was applied in our study aiming to further improve the selection efficiency of the Swedish Arctic charr strain. We inferred genomic relationships among the genotyped animals using a SNP derived genomic relationship matrix. The average genomic relationship among full sibs in our study was found to be 0.42 (Figure 1) which is lower than the expected theoretical value. In general, the average relationship among full sibs in the absence of inbreeding is expected to be 0.50. Prior application of low coverage GBS in selectively bred Atlantic salmon using varying SNP densities (∼24K – 30K) estimated an average relationship ranging between 0.36 - 0.45 among full sibs (Dodds *et al.* 2015). In comparison, an average genomic relationship of 0.47 was found among full sibs in selected lines of poultry through the usage of a 60K SNP array (Lourenco *et al.* 2015). Therefore, our study has likely underestimated the realized genomic relationships. Moreover, it has to be pointed out that when the genomic relationship matrix is constructed using allele frequencies estimated from the genotyped animals (as is the case in the current study) and not from the founders, genomic relationships seem to be underestimated (Legarra 2018). Overall though, the fact that the obtained distribution of genomic relationships did not exhibit long tails (typical pattern in the presence of genotyping or pedigree errors) suggests that genotyping through low coverage GBS in Arctic charr is efficient in providing insights regarding the realized genomic relationships among selection candidates and allowing therefore the exploitation of within family variance for selection purposes.

### Genetic diversity

As already mentioned, efficient management of inbreeding accumulation is of the utmost importance for the long-term sustainability of any breeding program. Therefore, genomic information can be particularly useful for guiding selection decisions that would minimize the loss of genetic diversity as has been already demonstrated in salmonids among other species (Barría *et al.* 2019). Even though inbreeding accumulation in the Arctic charr breeding program has been below 1% per generation according to available pedigree data, genomic tools can provide valuable insight regarding the existing genetic diversity and inform for optimal crosses among selection candidates.

The obtained heterozygosity metrics in our study showed that the H_e_ was lower than the H_o_ indicating an excess of heterozygotes which at first sight should correlate positively with the existence of ample genetic variation. In general, a breeding program would be expected to be more resilient to the loss of genetic diversity in comparison to commercial hatcheries where no pedigree records are kept. Even though our dataset was comprised only of related individuals derived from a closed breeding nucleus (particularly in the case of year class 2017 where the studied dataset was comprised of a small number of full-sib families) the obtained heterozygosity values were higher in comparisons to previous studies where we used GBS type methodologies in studying the diversity of farmed tilapia populations from commercial hatcheries with no pedigree recordings (Kajungiro *et al.* 2019; Mbiru *et al.* 2019).

Nevertheless, it would seem premature to infer that due to the excess of heterozygotes the existing genetic diversity in the Arctic charr breeding program is sufficient. Interestingly, according to literature an excess of heterozygotes has been observed in small and relatively recently founded populations of both animals and plants with separate sexes (Allendorf and Luikart 2007). Naturally, the above phenomenon would be ephemeral, since in the long-term heterozygosity would eventually decline. The background history of the Arctic charr breeding program suggests that the founder population consisted of a small number of fish (Eriksson *et al.* 2010). It has to be stressed, that a potential pitfall of the current study lies on the fact that the methodology for obtaining heterozygosity metrics through low coverage GBS is still under development. However, available data from high coverage ddRAD from a different subset of 2017 year class Arctic charr, suggested observed and expected heterozygosity values in close accordance with the values we obtained after depth adjustment in the current study (H_o_ = 0.33, H_e_ = 0.34; unpublished data).

Despite the fact that the Arctic charr breeding program has been operational for almost 40 years, no prior study attempted to investigate the genetic diversity of the selected population. The F_ST_ metric is commonly applied in studying the genetic differentiation among populations (Cockerham and Weir 1984). Therefore, strictly on technical terms the current dataset is not expected to be highly informative for such analysis. Furthermore, no F_ST_ calculations were performed for the animals from year class 2013 since those animals constituted a representative sample of the breeding program for the aforementioned generation. However, F_ST_ comparisons can provide valuable insights regarding the differentiation of the genotyped families from year class 2017. In order to minimize the possibility of obtaining inflated F_ST_ values due to the reduced accuracy of genotype calling in low coverage GBS we estimated median F_ST_ values, since the latter are less sensitive to outliers (as a result of genotypic errors). The obtained comparisons among the genotyped families indicated low genetic differentiation among the tested families (0.009 – 0.066) which appears to be in line with the history of the breeding program.

Moreover, in the current study we attempted to study in higher depth the genetic differentiation among the tested animals using PCA and DAPC. Applying PCA to the animals from the 2013 year class did not indicate the existence of distinct clusters. On the other hand the corresponding application of PCA in animals from year class 2017 provided indications regarding the existence of distinct clusters. The underlying algorithm of PCA aims to summarize the total variation of the tested dataset in a reduced dimension. Furthermore, a major advantage of PCA is its computational efficiency. Nevertheless, the above approach is not optimal for distinguishing different clusters and PCA does not qualify strictly speaking as a clustering algorithm. DAPC on the other hand retains the computational advantages of PCA, but at the same time offers higher resolution for detecting genetic clusters (Jombart *et al.* 2010). In addition, being an unsupervised learning approach, DAPC has the potential of providing valuable genetic insight in samples of unknown origin. Over the years, genetic material from the Arctic charr breeding program has been disseminated in various farms across the country (Nilsson *et al.* 2010). Potential crossbreeding with other Arctic charr strains is therefore likely to have occurred in commercial farms. As such, DAPC could assist in distinguishing the selectively bred Arctic charr from other farmed strains or potential crossbreds in Sweden. DAPC was successful in identifying clusters that were in agreement with pedigree records. In particular, families originating from the same grandsire and granddam were indistinguishable (*e.g.*, families 5,7 and 9,10) and inseparable in comparison to other families that were more distantly related according to pedigree records. Overall, through GBS application informative decisions regarding selection crosses can be obtained in the future even in the absence of pedigree records.

### Parentage assignment

An additional advantage of genetic markers, most relevant for selective breeding, is their use for parentage assignment purposes. Unfortunately, in the current study it was not possible to obtain tissue samples from all the putative parents for the animals of the 2017 year class (only for 2 full sib families we genotyped both parents). The analysis conducted in the subset of families with at least one parent genotyped (8 families) demonstrated that the derived genomic information was sufficient for verifying putative parentage records. Apart from the obtained discrepancy between genotypes and pedigree for the putative dam of family 2, the obtained genotypic information verified the pedigree parentage records implying that low coverage GBS is an effective approach for parentage assignment as already demonstrated in red deer (Dodds *et al.* 2019). Despite the fact that genotype calling in low coverage GBS entails higher uncertainty compared with high coverage approaches (Dodds *et al.* 2015) all full-sibs of family 2 indicated the existence of a pedigree error for that particular dam. Furthermore, the relationship among the full sibs of family 2 was in the range we recorded for all tested full sib pairs (0.35 – 0.45). As such, the above results indicate that a pedigree recording error is the most likely explanation.

### Heritability for growth traits - accuracy of breeding value estimation

Moderate heritabilities (∼0.20) for juvenile growth-related traits were obtained in the current study suggesting that ample genetic variance exists for further improving the Swedish Arctic charr strain. A high common full-sib effect was found (0.11) which might be confounding with the additive genetic variance. As such the obtained heritability might be underestimated. Nevertheless, it has to be pointed out that a small number of families (13) was used in the current study. In addition, the number of animals genotyped per family varied widely (16 – 90). Therefore the obtained heritability metrics should be treated with caution.

Even though genomic selection through GBS related approaches has proven to consistently outperform pedigree BLUP in aquaculture species (Barría *et al.* 2018; Aslam *et al.* 2018; Kyriakis *et al.* 2019) no prior study attempted to investigate the efficiency of low coverage GBS on prediction accuracy in farmed fish. The results from the genomic prediction approach were encouraging for a future practical implementation of GBLUP in the selected strain. The inclusion of genomic relationships resulted in an improved breeding value estimation accuracy clearly outperforming the corresponding pedigree BLUP based model. Additionally, our results appear to be in accordance with prior simulation derived data where low coverage GBS strategies were considered promising for delivering high prediction accuracies as opposed to traditional pedigree based approaches (Gorjanc *et al.* 2015). Nevertheless, we would have to acknowledge that the small dataset we used for genomic prediction (759 offspring from year class 2017) limits our ability of drawing definite conclusions.

## Conclusions

Overall, low coverage GBS has been proven to be an efficient and cost-effective approach for obtaining a wide range of essential information for selective breeding purposes. Applying low coverage GBS in the selected Arctic charr strain allowed us to gain insightful information regarding the genetic diversity of the stock, verify (or reject) parentage records and estimate genomic breeding values. Further studies focusing on application of GBS in studying resistance of Arctic charr to common encountered diseases would be deemed particularly promising since it would be possible to exploit the within family variance during selection. Finally, studies aiming to combine low coverage GBS with single-step BLUP approaches (Aguilar *et al.* 2011; Legarra *et al.* 2014) where only a subset of the selection candidates is genotyped, could be particularly valuable regarding cost efficiency.
